# Antiphospholipase A_2_ Receptor Autoantibodies: A Comparison of Three Different Immunoassays for the Diagnosis of Idiopathic Membranous Nephropathy

**DOI:** 10.1155/2014/143274

**Published:** 2014-04-09

**Authors:** Astrid Behnert, Mario Schiffer, Janina Müller-Deile, Laurence H. Beck, Michael Mahler, Marvin J. Fritzler

**Affiliations:** ^1^Division of Nephrology, Hannover Medical School, Carl-Neuberg-straße, 30625 Hannover, Germany; ^2^Section of Nephrology, Department of Medicine, Boston University School of Medicine, Boston, MA 02118, USA; ^3^INOVA Diagnostics, INC. 9900 Old Grove Road, San Diego, CA 32131-1638, USA; ^4^Faculty of Medicine, University of Calgary, 3330 Hospital Dr. NW, Calgary, AB, Canada T2N 4N1

## Abstract

*Background*. The recent identification of circulating autoantibodies directed towards the M-type phospholipase A_2_ receptor (PLA_2_R) has been a major advancement in the serological diagnosis of idiopathic membranous nephropathy (IMN), a common cause of nephrotic syndrome in adults. The goal of this study was to compare the performance characteristics of two commercial assays as well as the first addressable laser bead immunoassay (ALBIA) developed for the detection of anti-PLA_2_R antibodies. *Methods.* Serum samples of 157 IMN patients and 142 controls were studied. Samples were tested by a cell based immunofluorescence assay (CBA-IFA, Euroimmun, Germany), by ELISA (Euroimmun), and by a novel ALBIA employing an in vivo expressed recombinant human PLA_2_R. *Results*. Overall, the three assays showed significant qualitative and quantitative correlation. As revealed by receiver operating characteristic analysis, the ALBIA correlated better with the CBA-IFA than the ELISA (*P* = 0.0003). The clinical sensitivities/specificities for IMN were 60.0% (51.0–68.5%)/98.6% (95.0–99.8%) and 56.2% (47.2–64.8%)/100.0% (97.4–100.0%) for ALBIA and CBA-IFA, respectively. *Conclusion*. The ALBIA represents a promising assay for the detection of anti-PLA_2_R antibodies showing similar performance to the CBA-IFA and the advantage of ease of use and suitability for high throughput, rapid turnaround times, and multiplexing.

## 1. Introduction

Idiopathic membranous nephropathy (IMN) is a common cause of nephrotic syndrome in adults and has been identified as an autoimmune-mediated disease [1–3]. A number of studies have shown that 52–82% of IMN sera have autoantibodies directed towards the M-type phospholipase A_2_ receptor [4–6], a 180 kDa protein that is expressed by alveolar epithelial cells and neutrophils but is mainly restricted to podocytes within the kidney. Autoantibodies directed to PLA_2_R are fairly specific for primary or idiopathic MN but are also found in only a small proportion of sera from patients with secondary MN [4]. Accordingly, the detection of anti-PLA_2_R antibodies helps to differentiate between primary and secondary MN and other autoimmune nephropathies that may present with similar clinical features [7–9]. In addition, a positive test may be used in conjunction with clinical features to indicate a need for immunosuppressive therapy and the autoantibody titers used to monitor patients during therapy [8, 9].

Until recently, the only commercially available immunoassay for determining anti-PLA_2_R antibodies has been a semiquantitative cell based assay utilizing indirect immunofluorescence (CBA-IFA). Although this assay is relatively inexpensive and easy to perform, it is not well suited to high throughput laboratories and can be troubled by subjective interpretation. Recently, we reported a quantitative, observer-independent, high throughput immunoassay on an addressable laser bead immunoassay (ALBIA) platform that employed cell lysates bearing the full-length recombinant human protein to reliably detect anti-PLA_2_R antibodies in IMN sera [10]. ALBIA is a multiplexing laser bead technology in which specific autoantigens are covalently coupled to microspheres labeled internally with different ratios of two fluorochromes. After incubation with human sera and a fluorochrome (i.e., phycoerythrin) conjugated secondary antibody, beads are analyzed with two lasers. One laser is used to detect the “color” of the bead bearing the coupled antigen of interest (i.e., PLA_2_R), while the second laser is used to determine the binding of fluorochrome-coupled secondary antibody bound to the target antigen-autoantibody complex on the bead [11, 12]. The fluorescence intensity is digitally interpolated and expressed as median fluorescence intensity (MFI). The ALBIA offers simultaneous testing for multiple targets in a single assay and requires only small serum sample volumes of 2–20 *μ*L. More recently, an ELISA based on purified human recombinant PLA_2_R extracted from transfected cells has been developed by the same company that manufactured the CBA-IFA [13, 14]. The objective of this study was to compare the novel ALBIA with CBA-IFA and ELISA for the detection of anti-PLA_2_R antibodies.

## 2. Material and Methods

### 2.1. Patients and Samples

Patient serum samples were collected at the Medical School Hannover, Germany, and Boston University School of Medicine, MA, USA. The study included sera from 157 IMN patients, whose diagnosis was supported by typical biopsy features of primary MN without evidence of secondary features or clinical associations, as well as 50 normal healthy controls, 41 nephrotic disease controls (patients presenting with nephrotic syndrome in which biopsy revealed underlying cause different from IMN (see Supplementary Material available online at http://dx.doi.org/10.1155/2014/143274)), 26 systemic lupus erythematosus (SLE) patients and 25 patients with granulomatosis with polyangiitis (GPA; formerly Wegener's granulomatosis). Due to limitations in sample volume, not all samples were tested by all methods. Clinical data of the IMN cohort is provided in the Supplementary Material. Our study had a cross-sectional design and the serum samples were mainly obtained at the time of first consultation by a nephrologist. Therefore, the majority of patients in our study cohort has nephrotic range proteinuria and is either untreated or treated with renin-angiotensin-aldosterone system (RAAS) inhibitors only. Less than 10% of the serum samples were taken at follow-up visits where the patients were already undergoing specific immunotherapy. This study was approved by Ethics Committee of Medical School Hannover, Germany (Nr: 1246–2011) and patient data were anonymously used according to the latest version of the Helsinki Declaration of Human Research Ethics.

### 2.2. Immunoassays

All samples were tested by CBA-IFA (Euroimmun, Luebeck, Germany and Euroimmun, USA) and ALBIA (Mitogen Advanced Diagnostics Laboratory, Calgary, Canada). Samples from IMN patients were also tested by ELISA (Euroimmun, Luebeck, Germany and Euroimmun, USA) according to the manufacturer's protocol. The ALBIA was performed as previously described [10]. In brief, microbeads (Luminex, Austin, TX, USA) were indirectly coupled with the overexpressed full-length PLA_2_R captured from HEK cell lysates and incubated with diluted serum. PE conjugated anti-human IgG (Jackson ImmunoResearch, West Grove, PA, USA) was then added and after incubation the reactivity of individual sera was analyzed using a Luminex-100 luminometer (Luminex) and the MFI recorded. The ALBIA MFI cut-off value was calculated from receiver operating characteristics (ROC) curve analysis. ELISA and CBA-IFA cut-off values were established according to manufacturer's protocol (ELISA cut-off: 20 RU; CBA-IFA: negative versus 1 : 10 dilution; if positive at a dilution of 1 : 10: titration to final titer).

### 2.3. Statistical Analysis

The data was statistically evaluated using the Analyse-it software (Version 1.62; Analyse-it Software, Ltd., Leeds, UK). Chi-square, Spearman's correlation, and Cohen's kappa agreement tests were carried out to analyze the agreement between portions and *P* values < 0.05 were considered significant. ROC analysis was used to analyze the discriminatory ability of different immunoassays.

## 3. Results

### 3.1. Qualitative and Quantitative Agreements

**Figure 1:**
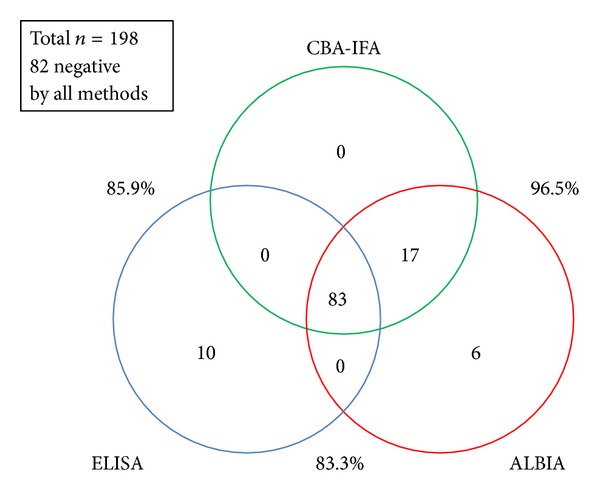
Correlation between different diagnostic immunoassays for the detection of anti-PLA_2_R antibodies. In our cohort of 157 IMN patients and 41 nephrotic disease control patients, a total of 83 samples were positive and 82 were negative by all three methods, a concordance of 77.6% for all three methods. Overall qualitative agreements were 85.9% for CBA-IFA versus ELISA, 96.5% for CBA-IFA versus ALBIA, and 83.3% for ELISA versus ALBIA.

Using the cut-off values established by the manufacturer for the ELISA and in our previous study for the ALBIA, good qualitative agreements were found (see Table 1). The overall qualitative agreements were 85.9% (95% confidence interval: 80.2–90.4%) for CBA-IFA versus ELISA, 96.5% (95% CI 92.9–98.6%) for CBA-IFA versus ALBIA, and 83.3% (95% CI 77.4–88.2%) for ELISA versus ALBIA. Venn diagram analysis showed that 83 samples were positive and 82 negative by all three methods. Overlap and discordance of the individual methods are illustrated in Figure 1.

**Figure 2:**
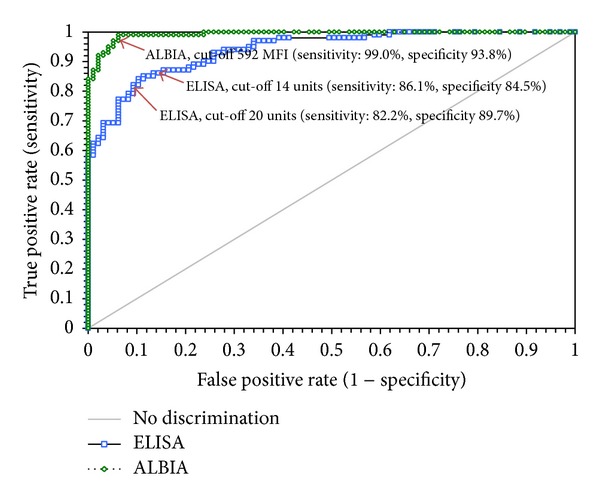
Comparative receiver operating characteristic (ROC) analysis (CBA-IFA positive versus negative samples). The ALBIA and the EUROIMMUN ELISA were compared to the EUROIMMUN CBA-IFA. The EUROIMMUN CBA-IFA was the first commercially available immunoassay for anti-PLA_2_R and therefore was used to define the outcome (anti-PLA_2_R positive versus anti-PLA_2_R negative). With an area under the curve (AUC) of 0.99 (95% CI: 0.99 to 1.00), the ALBIA performed similar to the CBA-IFA assay. The ELISA reached an AUC of 0.94 (95% CI: 0.91 to 0.97). Cut-off values are indicated by the arrows. The ELISA was also analyzed with an alternative (borderline) cut-off of 14 units.

Good qualitative agreements were also observed. The Spearman rho values were 0.75 (95% 0.67–0.81) for ALBIA versus ELISA, 0.79 (95% 0.73–0.84) for ELISA versus CBA-IFA, and 0.85 (95% 0.81–0.89) for ALBIA versus CBA-IFA. Using ROC analyses with the CBA-IFA results as the comparator, excellent discrimination was found for ALBIA and good discrimination for ELISA (see Figure 2). Area under the curve values were 0.99 (95% CI: 0.99 to 1,00) for ALBIA and 0.94 (95% CI: 0.91 to 0.97) for ELISA. The difference between the two AUCs was significant (*P* = 0.0003). The analysis was also done with the alternative (borderline) ELISA cut-off of 14 units leading to a higher sensitivity (Figure 2).

### 3.2. Clinical Performance Evaluation

**Figure 3:**
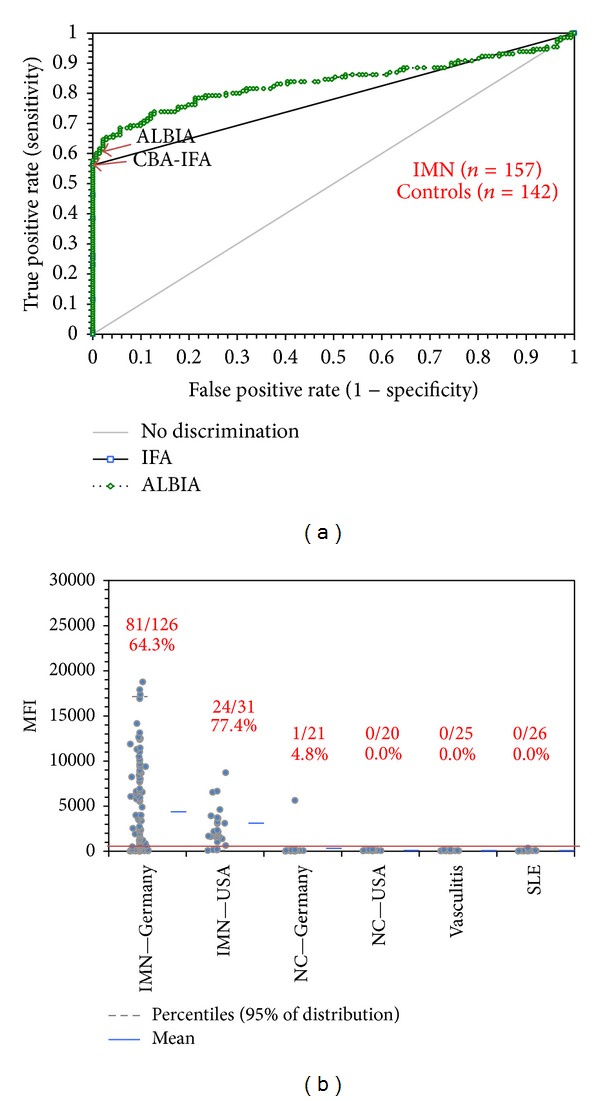
Comparative analysis (IMN versus controls). ALBIA and the CBA-IFA were compared to the diagnosis of the subjects tested. (a) With an area under the curve (AUC) of 0.78 (95% CI: 0.74–0.82) for CBA-IFA and of 0.84 (95% CI: 0.78–0.89) for ALBIA, both assays showed similar discrimination between IMN and controls. Cut-off values are indicated by the arrows. (b) Comparative descriptive analysis shows the prevalence of anti-PLA_2_R antibodies in different cohorts measured by ALBIA.

In our cohort of 198 patients with nephrotic syndrome tested by all three methods, 100 (50.51%) were positive and 98 negative for anti-PLA_2_R antibodies by CBA-IFA. In the recently released ELISA, 93 (46.97%) were positive and 105 negative. Lastly, 106 (53.54%) were positive and 92 negative by ALBIA. Since the ALBIA demonstrated significantly better agreement with CBA-IFA, we focused the specificity study on ALBIA and CBA-IFA. In the clinical performance study, ROC analysis showed similar discrimination between IMN patients and various controls. With an area under the curve (AUC) of 0.78 (95% CI: 0.74–0.82) for CBA-IFA and of 0.84 (95% CI: 0.78–0.89) for ALBIA, both assays show similar discrimination between IMN and controls (see Figure 3(a)). No significant difference was observed in the prevalence of anti-PLA_2_R antibody positive samples among two different clinical sites (see Figure 3(b)). Performance characteristics are summarized in Table 2.

## 4. Discussion

The early diagnosis, differentiation from other nephropathies, and appropriate clinical management of IMN have been significantly improved by the detection and quantification of anti-PLA_2_R antibodies [7, 9, 15]. Here we compared two commercially available immunoassays as well as our in-house, research-based ALBIA for their accuracy. The ALBIA that is now routinely used as part of our research efforts correlated better with the CBA-IFA than the commercially available ELISA. Our data which found that the correlation of ELISA versus CBA-IFA was 0.79 (95% CI: 0.73–0.84) is similar to a recent study that found that a correlation of 0.75 (95% CI: 0.72–0.76) [13]. It is worth noting that, based on our observations, we believe that sensitivity, specificity, and concordance of the ELISA with the other immunoassays increase when a lower RU cut-off is applied.

The differences in the performance of the different assays might be explained by the differences in the antigen binding matrices utilized in these platforms. In ELISA, antigens are passively absorbed to the plastic matrix and reactivity of antibodies is highly dependent on sufficiently exposed epitopes available for binding or not sterically restricted for antibody binding [16]. By comparison, in ALBIA, the antigen is covalently linked to spherical beads in suspension which may facilitate binding of autoantibodies to the cognate, more sterically accessible conformational epitopes. In the CBA-IFA, recombinant PLA_2_R is overexpressed in transfected HEK cells and the protein presumably targets to its native cellular domain allowing a more “native” expression of epitopes.

The ALBIA is a high throughput immunoassay requiring only a small amount of serum. In addition, the multiplex format is designed to simultaneously measure multiple targets including cytokines, complement, and antibodies in each sample and extremely flexible in the combination of assays to multiplex. It allows testing for many differential diagnoses (e.g., granulomatosis with polyangiitis, Goodpasture's disease, IMN, lupus nephritis, etc.) at a single time and to facilitate more accurate diagnosis [11].

In the present study, we investigated the correlation and agreement between three different immunoassays for the detection of anti-PLA_2_R antibodies in IMN patients. Therefore, our study allowed for the assessment of clinical sensitivity. Although the three assays perform similarly, a significant limitation of the CBA-IFA is the adaptability to high throughput laboratories where diagnostic platforms such as ELSIA and ALBIA might be preferred. This is the first reported ALBIA developed for the detection of anti-PLA_2_R antibodies and it had good comparative performance to CBA-IFA. Since the ALBIA platform is easily adopted to high throughput testing and rapid turnaround times, it might be considered for future commercial assay development. Additionally, CBA-IFA is semiquantitative and, in a clinical setting where anti-PLA_2_R antibodies might be used to monitor treatment response and disease progression [7, 9, 14], quantitative ELISA and ALBIA would have an advantage because they provide a more accurate reflection of changes in the antibody titers. Finally, the multiplex capability of ALBIA offers opportunities to develop autoantibody, histocompatibility, immunoglobulin isotype, cytokine, and complement panels [11, 12] that aid in the differential diagnosis of autoimmune kidney diseases.

## Supplementary Material

Clinical characteristics of IMN patients and Nephrotic Disease Control patients are described in the first two tables and include gender distribution, age, serum-creatinin, serum-urea, serum-albumin and proteinuria. Characteristics were similar except for a male predominance in our IMN cohort and a greater proteinuria in our Nephrotic Disease Control cohort. The Nephrotic Disease Control cohort includes patients with various diagnoses as can be seen in the third table.

## Figures and Tables

**Table 1:** Qualitative agreements between the different methods.

All IMN and NC patients (*n* = 198)	IFA-CBA			Percent agreement (95% confidence)
	Positive	Negative	Total	
ELISA				
Positive	83	10	93	Pos. agreement = 82.2% (73.3–89.1%)
Negative	18	87	105	Neg. agreement = 89.7% (81.9–94.9%)

Total	101	97	198	Total agreement = 85.9% (80.2–90.4%)

*kappa* = 0.72 (95% CI 0.62–0.81)				

All IMN and NC patients (*n* = 198)	IFA-CBA			Percent Agreement (95% confidence)
	Positive	Negative	Total	

ALBIA				
Positive	100	6	106	Pos agreement = 99.0% (94.6–100.0%)
Negative	1	91	92	Neg agreement = 93.8% (87.0–97.7%)

Total	101	97	198	Total agreement = 96.5% (92.9–98.6%)

*kappa* = 0.93 (95% CI 0.88–0.98)				

All IMN and NC patients (*n* = 198)	ELISA			Percent agreement (95% confidence)
	Positive	Negative	Total	

ALBIA				
Positive	83	23	106	Pos agreement = 89.2% (81.1–94.7%)
Negative	10	82	92	Neg agreement = 78.1% (69.0–85.6%)

Total	93	105	198	Total agreement = 83.3% (77.4–88.2%)

*kappa* = 0.67 (95% CI 0.57–0.77)				

**Table 2:** Clinical sensitivity and specificity for CBA-IFA and ALBIA.

All patients and Controls (*n* = 299)	Samples			Percent agreement (95% confidence)
	IMN	Controls	Total	
CBA-IFA				
Positive	100	1	101	Sensitivity = 63.7% (55.7–71.2%)
Negative	57	141	198	Specificity = 99.3% (96.1–100.0%)

Total	157	142	299	

ALBIA				
Positive	105	2	108	Sensitivity = 66.9% (58.9–74.2%)
Negative	52	139	191	Specificity = 97.9% (94.0–99.6%)

Total	157	142	299	
