# Supplementary material for: Antiphospholipase A2 Receptor Autoantibodies: A Comparison of Three Different Immunoassays for the Diagnosis of Idiopathic Membranous Nephropathy
Source: J Immunol Res. 2014 Apr 9;2014:143274. doi: 10.1155/2014/143274 (PMC4000632; doi:10.1155/2014/143274)
Supplement: Supplementary file 1 — Clinical characteristics of IMN patients and Nephrotic Disease Control patients are described in the first two tables and include gender distribution, age, serum-creatinin, serum-urea, serum-albumin and proteinuria. Characteristics were similar except for a male predominance in our IMN cohort and a greater proteinuria in our Nephrotic Disease Control cohort. The Nephrotic Disease Control cohort includes patients with various diagnoses as can be seen in the third table. [file 143274.f1.zip › 143274.f1/143274.f4.docx]

**Supplementary Material**

**Clinical data of IMN cohort:**

| **n** | 157 |
| --- | --- |
| **Gender (m:w)** | 126:31 |
|  | ***Mean (95% CI)*** |
| **Age** | 53.26 (50.65 - 55.87) |
| **S- Creatinin (µmol/l)** | 127.63 (116.88 - 138.88) |
| **S- Urea (mmol/l)** | 11.11 (9.76 - 12.47) |
| **S- Albumin (g/l)** | 29.09 (27.57 - 30.60) |
| **Proteinuria (mg/g Krea)** | 4442 (3674 - 5209) |

**Clinical data of Nephrotic Disease Control cohort:**

| **n** | 41 |
| --- | --- |
| **Gender (m:w)** | 21:20 |
|  | ***Mean (95% CI)*** |
| **Age** | 44.34 (39.11 - 49.57) |
| **S- Creatinin (µmol/l)** | 93.59 (75.76 - 111.41) |
| **S- Urea (mmol/l)** | 9.15 (4.96 - 13.34) |
| **S- Albumin (g/l)** | 30.95 (27.01 - 34.88) |
| **Proteinuria (mg/g Krea)** | 6057 (4427 - 7687) |

**Nephrotic Disease Control cohort includes the following diagnoses:**

| **Diagnosis** | **#** |
| --- | --- |
| FSGS | 6 |
| Secondary MN | (15) |
| - Lupus | 3 |
| - HBV | 3 |
| - NSAIDs | 1 |
| - Mercury | 1 |
| - Rheumatoid Arthritis | 1 |
| - Other autoimmune disease | 2 |
| - n/a | 4 |
| Diabetic Nephropathy | 4 |
| IgA Nephropathy | 7 |
| Hypertensive Nephropathy | 1 |
| MPGN | 2 |
| Minimal Change Disease | 4 |
| Collapsing GN (viral syndrome) | 1 |
| Immune-complex GN of unknown etiology | 1 |
| ***total:*** | **41** |
