# Supplementary figures and images for: Antiphospholipase A2 Receptor Autoantibodies: A Comparison of Three Different Immunoassays for the Diagnosis of Idiopathic Membranous Nephropathy
Source: J Immunol Res. 2014 Apr 9;2014:143274. doi: 10.1155/2014/143274 (PMC4000632; doi:10.1155/2014/143274)

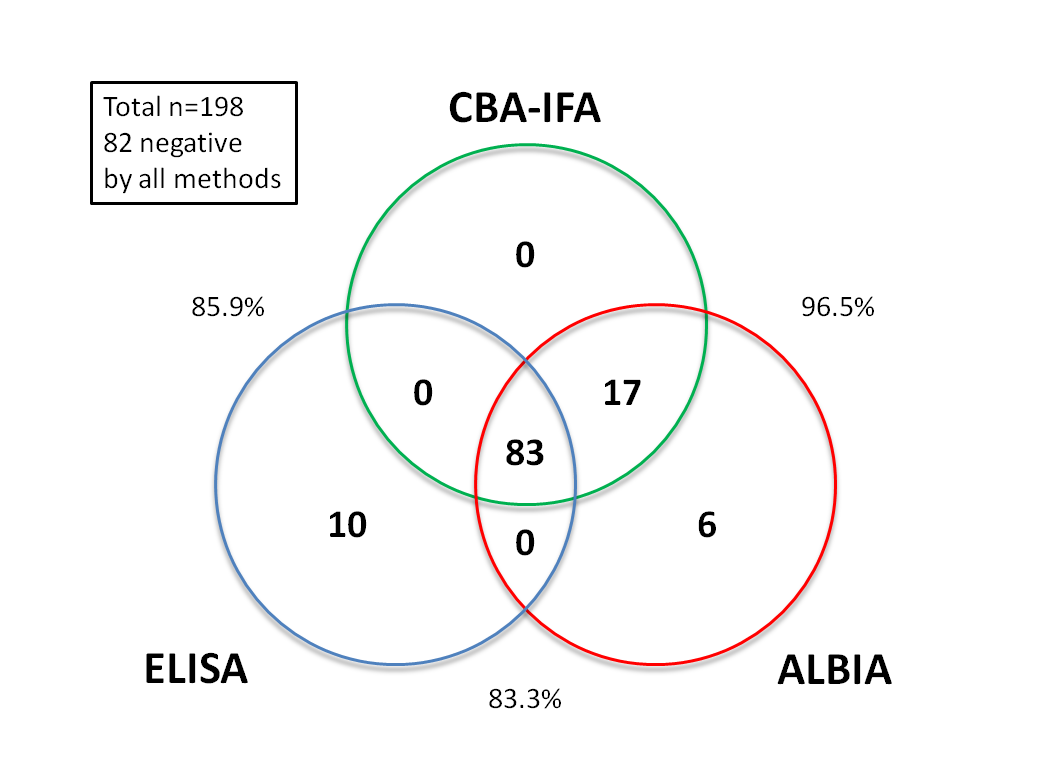

Supplement: Supplementary file 1 — Clinical characteristics of IMN patients and Nephrotic Disease Control patients are described in the first two tables and include gender distribution, age, serum-creatinin, serum-urea, serum-albumin and proteinuria. Characteristics were similar except for a male predominance in our IMN cohort and a greater proteinuria in our Nephrotic Disease Control cohort. The Nephrotic Disease Control cohort includes patients with various diagnoses as can be seen in the third table. [file 143274.f1.zip › 143274.f1/143274.f1.docx]

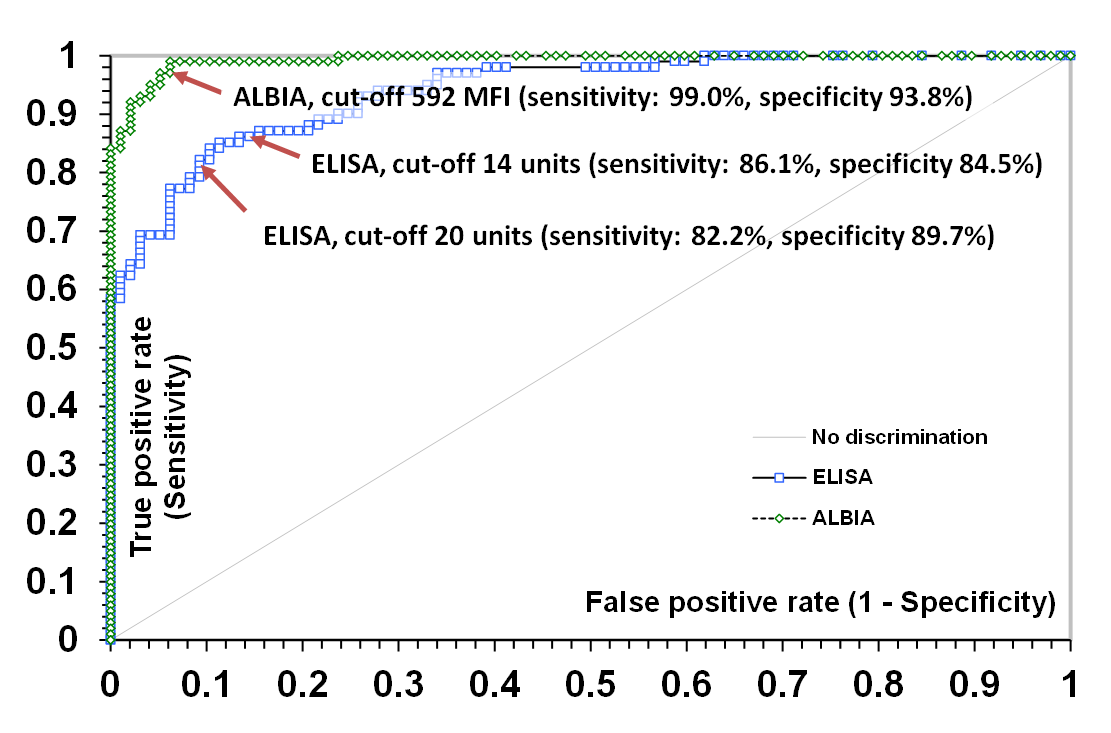

Supplement: Supplementary file 1 — Clinical characteristics of IMN patients and Nephrotic Disease Control patients are described in the first two tables and include gender distribution, age, serum-creatinin, serum-urea, serum-albumin and proteinuria. Characteristics were similar except for a male predominance in our IMN cohort and a greater proteinuria in our Nephrotic Disease Control cohort. The Nephrotic Disease Control cohort includes patients with various diagnoses as can be seen in the third table. [file 143274.f1.zip › 143274.f1/143274.f2.docx]

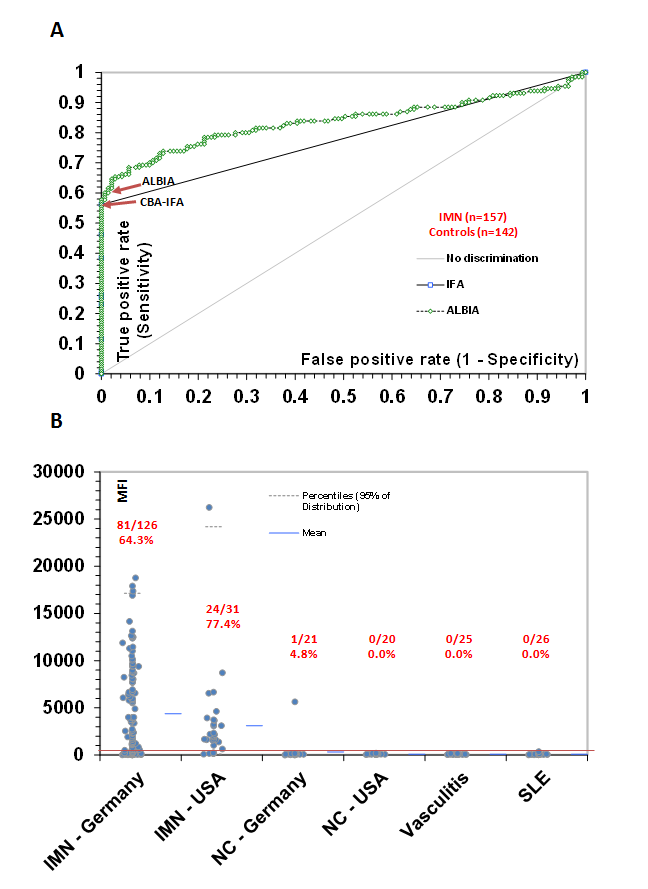

Supplement: Supplementary file 1 — Clinical characteristics of IMN patients and Nephrotic Disease Control patients are described in the first two tables and include gender distribution, age, serum-creatinin, serum-urea, serum-albumin and proteinuria. Characteristics were similar except for a male predominance in our IMN cohort and a greater proteinuria in our Nephrotic Disease Control cohort. The Nephrotic Disease Control cohort includes patients with various diagnoses as can be seen in the third table. [file 143274.f1.zip › 143274.f1/143274.f3.docx]
